# Investigation of the Effect of Hafnium Chloride on Sperm Viability and Motility in Normospermic Cases: An In Vitro Study

**DOI:** 10.7759/cureus.62084

**Published:** 2024-06-10

**Authors:** Zülkar Özden, Fatma Öz Bağcı, Gülsemin Çiçek, Tahsin Murad Aktan, Selçuk Duman

**Affiliations:** 1 Histology and Embryology, Rize Training and Research Hospital, Rize, TUR; 2 Histology and Embryology, Meram Faculty of Medicine, Necmettin Erbakan University, Konya, TUR

**Keywords:** toxicology, sperm viability, sperm motility, hafnium chloride, semen analysis

## Abstract

Introduction: Hafnium alloys are employed in medical applications due to their biocompatibility and high corrosion resistance. These alloys have demonstrated osteogenic and antimicrobial activities in surgical implants and have been utilized in the treatment of sarcoma. Additionally, a sensor based on hafnium nanoparticles has been reported for the detection of coronavirus disease 2019. Despite the increasing usage of hafnium, a literature review reveals no studies examining its effects on sperm in both human and animal species.

Methods: Semen samples were analyzed according to the 2010 World Health Organization (WHO) criteria, and 20 normospermic specimens were included in the study. Three groups were formed: control, hafnium chloride 2 mg/mL, and 4 mg/mL. Motility and viability were assessed in all groups at the 20th and 40th minutes.

Results: The decrease in viable sperm count was found to be significant in the 2 mg/ml HfCl_4_ group (difference: 12.73 ± 0.8, p<0.001) and the 4 mg/ml HfCl_4_ group (difference: 41.72 ± 1.34, p<0.001) compared to the control group. A time-dependent decrease in sperm viability was significant across all groups (difference: 8.93 ± 0.59, p<0.001). The decrease in viable sperm count in the 4 mg/ml HfCl_4_ group was significant when compared to the 2 mg/ml HfCl_4_ group (difference: 29 ± 1.27, p<0.001). The decrease in total motile sperm count was observed in both the 2 mg/ml HfCl_4_ group (difference: 12.80 ± 1.30, p<0.001) and the 4 mg/ml HfCl_4_ group (difference: 35.63 ± 1.12, p<0.001) compared to the control group. Additionally, the decrease in total motile sperm count in the 4 mg/ml HfCl_4_ group was significant compared to the 2 mg/ml HfCl_4_ group (difference: 22.80 ± 1.60, p<0.001). A time-dependent decrease in total motile sperm count was also significant (difference: 6.03 ± 0.49, p<0.001).

Conclusion: The study determined that hafnium chloride negatively affects sperm motility and viability in vitro. These effects may be due to the presence of an acidic environment. It has been demonstrated that instruments containing this element may pose a potential risk.

## Introduction

Hafnium is a transition element with an atomic number of 72 and an atomic weight of 178.6. It belongs to group IV B of the periodic table, alongside titanium and zirconium. In nature, hafnium is commonly found together with zirconium, with minerals containing zirconium typically comprising 1-4% hafnium. Hafnium and zirconium share very similar chemical and physical properties [1]. Almost all hafnium metals are produced through the reduction of hafnium tetrachloride using sodium or magnesium in a process known as the Kroll process. Hafnium tetrachloride is a white, monoclinic crystalline solid with the molecular formula HfCl_4_ and a molecular weight of 320.3. It is known to volatilize at approximately 250 °C [2].

Hafnium has been found to play a crucial role between silicate layers in maintaining high electron mobility as high-k dielectrics (insulators) in the production of integrated circuits [3]. Hafnium oxide provides high sensitivity in electrical sensors, and its chemical and thermodynamic stability makes it a very durable and long-lasting material, making hafnium oxide sensors superior to silicon oxide sensors. It has been emphasized that it can be used in medical diagnostics and food analysis [4]. Hafnium oxide has also been utilized in lithography, where it is dispersed in water [5]. As a result of these applications, hafnium nanoparticles can be released into process fluids and the surrounding environment.

In another aspect, the use of hafnium is also increasing in the medical field. Hafnium alloys have been demonstrated to be suitable for medical implants due to their biocompatibility and high corrosion resistance [6]. Hafnium nitride-based coatings for medical implants and instruments have been found to inhibit pathogenic microflora. In one study, hafnium nitride was added to 3D prints and demonstrated an antibacterial effect, particularly against Escherichia coli [7]. Hafnium nitride-based coating on the skin has been shown to inhibit the growth of pathogenic microflora without impairing the properties of the skin, especially its permeability and elasticity [8]. In vivo studies have shown the biocompatibility and osteogenic effects of hafnium [9,10]. Hafnium oxide microneedles have been utilized in transdermal drug applications [11]. Zirconium coatings in dental implants have become preferred in recent years due to their durability and biocompatibility. One study identified the presence of hafnium at a concentration of 2% in zirconium dental veneers [12]. A sensor based on hafnium nanoparticles has been reported for the detection of coronavirus disease 2019 (COVID-19), demonstrating high sensitivity and 100% specificity in 100 COVID-19-positive samples [13]. Consequently, the use of hafnium alloys is increasing day by day.

Studies on the toxicity of hafnium are quite limited. Haley et al. determined the intraperitoneal lethal dose (LD50) of hafnium chloride as 112 mg/kg in their study on rats [14]. Another study found hafnium oxide to be relatively non-toxic in HaCaT skin cells. Hafnium oxide solution caused a 50% response at 2200 mg/L in the live/dead test and a 50% response at 300 mg/L in the mitochondrial toxicity test [15].

Currently, the number of couples experiencing infertility is rising, with male factors contributing to 30-40% of infertility cases. Semen quality is a crucial indicator of male reproductive health, and semen analysis plays a critical role in andrology [16,17]. The decline in male reproductive potential can be congenital or acquired due to various factors such as urogenital or genetic anomalies, varicocele, genital system infections, endocrine disorders, cancer, and exposure to gonadotoxic agents [18].

Decreased fertility rates have been associated with environmental toxic exposures, including heavy metals [19-21]. Phthalates, which are commonly used in plastics, have been linked to infertility and decreased sperm quality [22]. Motor vehicle exhaust smoke, a significant cause of air pollution, has been shown to reduce sperm quality [23]. Dioxins, by-products of forest fires, commercial waste incineration, pesticides, and paper production have negative effects on sperm motility, morphology, and count [24]. Environmental exposure to metals and metalloids such as arsenic (As), cadmium (Cd), copper (Cu), selenium (Se), and tin (Sn) has been reported to impair male reproductive health [25]. Cadmium, a heavy metal ingested through smoking and food, negatively impacts spermatogenesis, particularly sperm motility [26]. A previous study indicated that transition elements such as cadmium, copper, molybdenum, and zinc had the highest concentrations in the semen of infertile men [27]. Although metals and metalloids are increasingly detected in humans, measuring these elements in seminal plasma rather than blood more accurately reflects the exposure of the male reproductive system [28,29]. As hafnium, a transition element, is increasingly used and negatively affects sperm motility and viability, detecting its presence in semen could be useful for understanding infertility and other tissue pathologies.

In assisted reproductive technologies, viability and sperm motility provide significant insights into sperm quality. The World Health Organization also prioritizes viability and motility values when assessing healthy semen parameters in terms of individuals' reproductive potential. Additionally, sperm motility and viability are more quickly affected by acute events in seminal plasma. For these reasons, our study focused on examining sperm motility and viability.

Considering the gap regarding the toxic potential of hafnium on reproduction, this review was written to provide current information regarding the potential adverse effects of hafnium on human reproduction. We also aimed to evaluate potential effects according to dose and exposure time.

This study has several strengths. First, to the best of our knowledge, this is the first study to evaluate the effects of hafnium chloride in seminal plasma. Second, we have identified the toxic dose of hafnium chloride in seminal plasma. This allowed us to assess the relationships between the level of hafnium chloride exposure and sperm quality indices in semen. Third, only individuals with normal sperm parameters were included in the study.

However, this study also has several limitations. First, an association has been identified between the presence of hafnium chloride in semen and sperm quality; however, a clear causality has not been established. Second, the participants in this study were recruited from the same reproductive center and underwent identical procedures.

This article was previously presented as a meeting poster at the 15th National - 1st International Congress of Histology and Embryology on May 26-28, 2022.

## Materials and methods

Study subjects and patient selection

Semen samples were collected from 20 male patients aged between 20 and 55 years with normal semen parameters according to the World Health Organization (WHO) 2010 criteria who applied to the Necmettin Erbakan University Meram Medical Faculty IVF Unit for routine semen analysis. The study was conducted with the approval of the Necmettin Erbakan University Meram Medical Faculty Non-Drug and Non-Medical Device Research Ethics Committee, dated July 17, 2020, and numbered 2020/2740. Informed written consent was obtained from all participants.

Preparation of hafnium chloride

Hafnium chloride (hafnium (IV) chloride, 98%, PN: 258202) used in the study was obtained from Sigma Aldrich, St. Louis, Missouri, USA. To prepare the stock HfCl_4_ solution, 2.5 g of HfCl_4_ was added to 10 mL of saline. The stock solution was stored at +4 °C and protected from light. Before each use, the stock solution was vortexed. The dosage was then adjusted to final concentrations of 2 mg/mL and 4 mg/mL HfCl_4_ in the washing medium. Based on our preliminary trials, we selected the hafnium concentration in the form of the minimum dose with toxic effects and the maximum dose without toxic effects.

Collection of semen samples

Semen samples were collected by masturbation after three days of sexual abstinence into sterile, non-toxic containers. The collected semen samples were allowed to liquefy at room temperature (23 °C) for 15-60 minutes. All subsequent procedures were performed at room temperature. Seminal plasma volume, liquefaction time, total sperm count, sperm concentration, sperm viability, and motility were measured according to WHO 2010 criteria. Based on these analyses, 20 normospermic semen samples were included in the study.

Preparation of semen samples

Normospermic semen was mixed 1:1 with sperm wash medium (Origio Sperm Wash, CooperSurgical®, Ballerup, Denmark) and centrifuged (Nüve, CN180, Turkey) at 150 g for 10 minutes. The supernatant was discarded, and 3 mL of sperm wash medium was added to the pellet and homogenized using an injector. The sample was then divided into three microcentrifuge tubes (Eppendorf, Hamburg, Germany), each containing 1 mL. The control group was given 16 µL of cell culture-grade water (Capricorn, Düsseldorf, Germany). HfCl_4_ stock solution was added to the other tubes to achieve final concentrations of 2 mg/mL and 4 mg/mL, respectively. Motility and viability were assessed at 20 and 40 minutes.

Motility determination

A 10 µL aliquot of the semen sample was placed in a Makler counting chamber. Two hundred cells were counted and evaluated under a microscope (Eclipse E200, Nikon Instruments, Inc., Tokyo, Japan) with a 20× objective. Sperm motility was divided into four groups according to WHO 2010 criteria. Progressive motility (PR) is sperm motility that moves in a linear or large circle. Non-progressive motility (NP) is defined by spermatozoa in which there is no progression in smaller circles, head movement is difficult or absent, and tail movement is observed. Immotility (IM) refers to spermatozoa without movement. The lower limit value of the percentage of progressive motile sperm (PR) is determined to be 32%, and the percentage of total motile sperm (PR+NP) is determined to be at least 40%. These parameters are important for infertility (WHO, 2010).

Viability determination

Viability was assessed using the eosin-Y stain. To prepare the solution, 0.9 g of sodium chloride (NaCl) was dissolved in 100 mL of pure water, and 0.5 g of eosin Y (color index: 45380) was added to 100 mL of 0.9% NaCl. For the viability test (WHO 2010), 10 µL of semen and 10 µL of eosin Y solution were mixed on a slide and covered with a coverslip. After two to three minutes, 200 spermatozoa were counted under a microscope with a 40× objective. Non-viable spermatozoa and immotile but viable spermatozoa are separated from each other by the presence of a cell membrane. Spermatozoa with damaged cell membranes absorb the dye. Spermatozoa that absorbed the dye were considered non-viable, while those that did not were considered viable. According to WHO 2010 criteria, the lower limit of viability is 58%.

pH measurement

pH test strips (Macherey-Nagel, Düren, Germany) were placed in a microcentrifuge tube (Eppendorf, Hamburg, Germany) until the color change stabilized. The color on the pH test strip was compared with the provided color scale.

Statistical analysis

At the conclusion of the study, motility and viability values for each group were recorded. Descriptive statistics, including the mean and standard deviation, were calculated for numerical variables. Mixed-effect models were used to analyze the data. All analyses were performed using Jamovi 2.0 software. A p-value of <0.05 was considered statistically significant.

## Results

As a result of the study, hafnium chloride significantly reduced sperm total motility, progressive motility, and viability. Additionally, it was determined that this effect increased further over time.

Total motility results

The decrease in total motile sperm count was observed in both the 2 mg/ml HfCl_4_ group (difference: 12.80 ± 1.30, p<0.001) and the 4 mg/ml HfCl_4_ group (difference: 35.63 ± 1.12, p<0.001) compared to the control group (Table 1). Additionally, the decrease in total motile sperm count in the 4 mg/ml HfCl_4_ group was significant compared to the 2 mg/ml HfCl_4_ group (difference: 22.80 ± 1.60, p<0.001). A time-dependent decrease in total motile sperm count was also significant across all groups (difference: 6.03 ± 0.49, p<0.001).

**Table 1:** Differences between groups Difference in sperm motility and viability values according to dose and time. SE: standard error.

Groups	Control: 2 mg/ml HfCl_4_		Control: 4 mg/ml HfCl_4_		2 mg/ml HfCl_4_ – 4 mg/ml HfCl_4_		Time		p-Value
	Difference	SE	Difference	SE	Difference	SE	Difference	SE	
Total motility %	12.80	1.30	35.63	1.12	22.80	1.60	6.03	0.49	<0.001
Progressive motility %	16.15	1.22	37.85	1.09	21.70	1.18	5.82	0.38	<0.001
Viability %	12.73	0.80	41.72	1.34	29.00	1.27	8.93	0.59	<0.001

**Figure 1:**
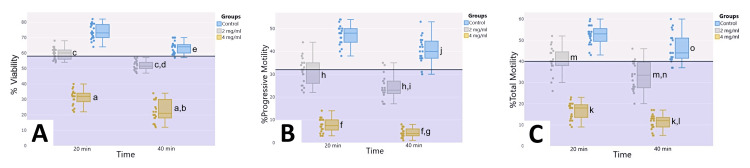
Illustration of the dose*time-dependent change in viability (A), progressive motility (B), and total motility (C) Illustration of the dose*time-dependent change in viability (A), progressive motility (B), and total motility (C) in the control and experimental groups according to WHO 2010 criteria. Each of the dots represents one sample. The dark blue line represents the lowest reference value of viability at 58% (A), progressive motility at 32% (B), and total motility at 40% (C). (A) Sperm viability: ^a^p<0.05, significantly different from control and 2 mg/mL HfCl_4_ group at 20 and 40 minutes; ^b^p<0.05, significantly different from 4 mg/mL HfCl_4_ group at 20 minute; ^c^p<0.05, significantly different from control group at 20 and 40 minutes; ^d^p<0.05, significantly different from 2 mg/mL HfCl_4_ group at 20 minutes; ^e^p<0.05, significantly different from control group at 20 minutes. (B) Sperm progressive motility: ^f^p<0.05, significantly different from control and 2 mg/mL HfCl_4_ group at 20 and 40 minutes; ^g^p<0.05, significantly different from 4 mg/mL HfCl_4_ group at 20 minutes; ^h^p<0.05, significantly different from control group at 20 and 40 minutes; ^i^p<0.05, significantly different from 2 mg/mL HfCl_4_ group at 20 minutes; ^j^p<0.05, significantly different from control group at 20 minutes. (C) Sperm total motility: ^k^p<0.05, significantly different from control and 2 mg/mL HfCl_4_ group at 20 and 40 minutes; ^l^p<0.05, significantly different from 4 mg/mL HfCl_4_ group at 20 minutes; ^m^p<0.05, significantly different from control group at 20 and 40 minutes; ^n^p<0.05, significantly different from 2 mg/mL HfCl_4_ group at 20 minutes;^ o^p<0.05, significantly different from control group at 20 minutes.

The total motility of spermatozoa in the control group (Table 2) remained within normal values (20 minutes: 52.40% ± 4.54%; 40 minutes: 46.65% ± 6.85%). In contrast, the percentage of total motile spermatozoa decreased significantly in both the 2 mg/mL HfCl_4_ (20 minutes: 40.20% ± 6.39%, p<0.05; 40 minutes: 33.25% ± 6.89%) and 4 mg/mL HfCl_4_ groups (20 minutes: 16.60% ± 3.97%, p<0.05; 40 minutes: 11.20% ± 3.58%). According to WHO 2010 criteria, total motility values in the 2 mg/mL HfCl_4_ group were mostly below the lowest reference value, while all values in the 4 mg/mL HfCl_4_ group were below the lowest reference value (Figure 1C).

**Table 2:** Mean and standard deviation values of motility and viability min: minute, SD: standard deviation, PR motility: progressive motility, NP motility: non-progressive motility.

Group	Control		2 mg/mL HfCl_4_		4 mg/mL HfCl_4_	
	Mean ± SD		Mean ± SD		Mean ± SD	
Time	20	40	20	40	20	40
Total motility %	52.4 ± 4.54	46.65 ± 6.85	40.2 ± 6.39	33.25 ± 6.89	16.6 ± 3.97	11.2 ± 3.58
PR motility %	47.25 ± 4.51	40.5 ± 5.62	31.4 ± 5.83	24.05 ± 5.2	7.7 ± 3.1	4.35 ± 1.98
NP motility %	5.15 ± 2.3	6.15 ± 2.16	8.8 ± 3.55	9.2 ± 4.61	8.9 ± 3.09	6.85 ± 2.89
Immotility %	47.6 ± 4.54	53.35 ± 6.85	59.8 ± 6.39	66.75 ± 6.89	83.4 ± 3.97	88.8 ± 3.58
Viability %	73.55 ± 4.99	63.45 ± 4.05	59.65 ± 3.82	51.9 ± 3.04	31.25 ± 4.84	22.3 ± 6.71

Progressive motility results

The decrease in progressive motile sperm count was observed in both the 2 mg/ml HfCl_4_ group (difference: 16.15 ± 1.22, p<0.001) and the 4 mg/ml HfCl_4_ group (difference: 37.85 ± 1.09, p<0.001) compared to the control group (Table 1). Additionally, the decrease in progressive motile sperm count in the 4 mg/ml HfCl_4_ group was significant compared to the 2 mg/ml HfCl_4_ group (difference: 21.70 ± 1.18, p<0.001). A time-dependent decrease in progressive motile sperm count was also significant across all groups (difference: 5.82 ± 0.38, p<0.001).

As shown in Table 2 and Figure 1B, progressive motility of spermatozoa in the control group remained within normal values (20 minutes: 47.25% ± 4.51%; 40 minutes: 40.50% ± 5.62%). In contrast, the percentage of progressive motile spermatozoa decreased significantly in both the 2 mg/mL HfCl_4_ (20 minutes: 31.40% ± 5.83%, p<0.05; 40 minutes: 24.05% ± 5.20%) and 4 mg/mL HfCl_4_ groups (20 minutes: 7.70% ± 3.10%, p<0.05; 40 minutes: 4.35% ± 1.98%). According to WHO 2010 criteria, the progressive motility values in the 2 mg/mL HfCl_4_ group were predominantly below the reference value, whereas all values in the 4 mg/mL HfCl_4_ group were consistently below the reference value (Figure 1B).

Viability results

The decrease in viable sperm count was significant in the 2 mg/ml HfCl_4_ group (difference: 12.73 ± 0.8, p<0.001) and the 4 mg/ml HfCl_4_ group (difference: 41.72 ± 1.34, p<0.001) compared to the control group (Table 1). A time-dependent decrease in sperm viability was significant across all groups (difference: 8.93 ± 0.59, p<0.001). The decrease in viable sperm count in the 4 mg/ml HfCl_4_ group was significant when compared to the 2 mg/ml HfCl_4_ group (difference: 29 ± 1.27, p<0.001).

In Table 2, the results indicate that the viability rate significantly decreased at 20 minutes in the 2 mg/mL HfCl_4_ (59.65% ± 3.82%, p<0.05) and 4 mg/mL HfCl_4_ (31.25% ± 4.84%, p<0.05) groups compared to the control group (73.55% ± 4.99%). Similarly, at 40 minutes, a significant decrease was observed in the 2 mg/mL HfCl_4_ (51.90% ± 3.04%, p<0.05) and 4 mg/mL HfCl_4_ (22.30% ± 6.71%, p<0.05) groups (Figure 1A) compared to the control group (63.45% ± 4.05%).

In Figure 1A, according to WHO 2010 criteria, the 20-minute viability values of the control group are above the lowest reference value. One sample at 40 minutes is within the lowest reference values. In the 2 mg/mL HfCl_4_ group, the 20-minute viability values started to fall below the lowest reference value. By the 40th minute, all samples were below the lowest reference value. In the 4 mg/mL HfCl_4_ group, all samples are below the lowest reference value.

**Figure 2:**
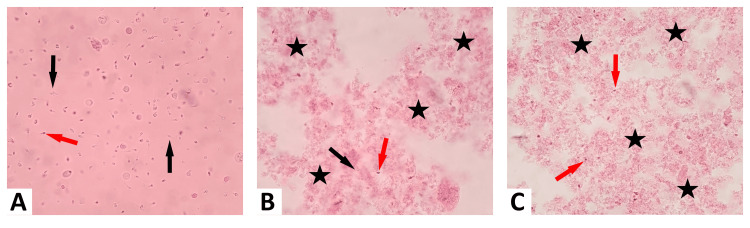
Eosin-Y staining Viable (black arrows), non-viable (red arrows) spermatozoa, and widespread sediment areas (asterisks) in the control group (A), and 4 mg/mL HfCl_4_ group (B, C) with eosin-Y staining at 20 (A, B) and 40 minutes (C). Diffuse precipitates (asterisks) were observed only in the 4 mg/mL HfCl_4_ group (B, C). Images (A-C) were obtained with 400× fiber optic magnification.

Figure 2A displays viable (black arrows) and non-viable (red arrows) spermatozoa in the control group at 20 minutes using eosin-Y staining. Figure 2B shows viable (black arrows) and non-viable (red arrows) spermatozoa and widespread sediment areas (asterisks) in the 4 mg/mL HfCl_4_ group at 20 minutes with eosin-Y staining. These precipitates are not observed in the control or 2 mg/mL HfCl_4_ groups. In Figure 2C, the 4 mg/mL HfCl_4_ group at 40 minutes shows non-viable (red arrows) spermatozoa and widespread sediment areas (asterisks). Diffuse precipitates were seen exclusively in the 4 mg/mL HfCl_4_ group.

According to the WHO 2010 criteria, there is a noticeable decrease in motility and viability values in the 2 mg/mL HfCl_4_ group, indicating a tendency away from normospermic values. The motility and viability values of the 4 mg/mL HfCl_4_ group fall significantly below the normospermic values (Table 2).

In addition to viability and motility values, pH was also measured in this study. The pH value of the control group was 8-8.5, as stated in the sperm-washing medium. We observed pH 6-7 in the 2 mg/mL HfCl_4_ group and pH 5 in the 4 mg/mL HfCl_4_ group.

## Discussion

This study revealed that HfCl_4_ decreased sperm motility and viability in vitro in a dose- and time-dependent manner. We found that motility and viability decreased the most in the 4 mg/ml HfCl_4_ group, especially in the 40th minute. Additionally, we found that as the dose of HfCl_4_ increased, the acidity also increased.

Huang et al. investigated the effect of metal ions iron ($\mathrm{Fe}_{2}^{+}$), manganese ($\mathrm{Mn}_{2}^{+}$), and lead ($\mathrm{Pb}_{2}^{+}$) on sperm motility at two, four, six, and eight hours and found a decrease in sperm motility as the concentrations of the elements increased [30]. It was concluded that $\mathrm{Fe}_{2}^{+}$ prevented sperm motility by lipid peroxidation, but $\mathrm{Mn}_{2}^{+}$ and $\mathrm{Pb}_{2}^{+}$ decreased sperm motility by an unknown mechanism. At the highest dose of 0.5 mg/mL and at the eighth hour, sperm motility decreased to 20% in the $\mathrm{Pb}_{2}^{+}$ and $\mathrm{Fe}_{2}^{+}$ groups and to 30% in the $\mathrm{Mn}_{2}^{+}$ group. In addition, the decrease in motility was more pronounced within the first two hours in both the control and element groups. In our study with hafnium chloride, we determined that the main effect occurs within the first 20 minutes. If they had made an assessment earlier, perhaps they could have achieved significant decreases. However, according to our results, later periods would have further reduced the decrease in mobility. The dose difference should also not be overlooked.

Zirconium, which has similar chemical properties to hafnium and contains low levels of hafnium, has been shown to damage the testes by increasing the production of reactive oxygen species. In the study conducted on rats, it was found that zirconium oxide nanoparticles disrupted spermatogenesis. In addition, narrowing of the seminal tubules and shrinkage of the lumen occurred [31]. Although there is no similar study on hafnium, the presence of hafnium in zirconium, its similar chemical properties, and the detection of its presence at a 2% ratio in zirconium dental crowns suggest that hafnium could have similar effects to zirconium or play a role in the effects produced by zirconium.

Haley et al. found that the intraperitoneal lethal dose (LD50) of hafnium chloride was 112 mg/kg. The symptoms of acute toxicity of hafnium chloride were lethargy and urination, and deaths usually occurred within the first 24 hours in mice. Histologically, vacuolization and granulation were observed in tissues in almost all animals. They stated that the acidic environment created by hafnium chloride has different effects from tissue to tissue. It was emphasized that the problems occurring in the skin and eyes were due to the acidic environment, while the cardiovascular collapse was not related to acidity [14]. In the study of Shelley on mice, a chondral mass was formed in the ear [32]. It has also been reported that hafnium can accumulate in different tissues in different animal species [33]. As discussed in these studies, hafnium can show different effects from tissue to tissue. In some tissues, it does not cause any effect, while in others, it causes serious side effects. The resulting side effects may differ due to the neutralization of the acidic environment produced by hafnium with body fluids. Although studies on the toxicity of hafnium chloride generally date back to before 2000, no studies on its effects on sperm have been reported.

Zhou et al. determined that low pH negatively affects sperm motility and viability. They observed a significant decrease in sperm motility and viability at acidic levels, particularly at pH 5.2 [34]. We found a decrease in sperm motility and viability in an acidic environment, as in the study of Zhou et al. The decreases in sperm motility in an acidic environment are similar. However, in the control groups, the decrease occurred at the 40th minute in our study, while it occurred at the 120th minute in their study. The contrast here may be due to the difference in the medium and method used during preparation and the use of computer-assisted semen analysis, or it may be due to the higher raw motility rates in their study. In addition, it should not be forgotten that the decline in sperm quality day by day makes sperm more sensitive to external factors. Although they used the HOS test for viability measurement, we used eosin Y staining and similarly found that viability decreased in acidic environments. In addition, we made a time-dependent comparison in the viability test and found that time-dependent viability decreased. As a result, it was determined that sperm motility and viability decreased in an acidic environment in both studies. The fact that the decreases in sperm parameters are close to each other brings to mind the question of whether hafnium chloride realizes its toxic effect only through acidic pH. In light of this limited information, hafnium chloride may have a toxic effect on sperm by causing an acidic environment.

In one study, zirconium hydroxide and hafnium hydroxide formed heavy precipitates below pH 7 and light precipitates above pH 7 [35]. In our study, diffuse precipitates were formed in the 4 mg/mL HfCl_4_ group with a pH value of 5. No precipitate was detected in the 2 mg/mL HfCl_4_ group with a pH value of 6-7. It has been emphasized that in order to minimize precipitates, the pH value should be above 7. In our study, although the pH was above 7, we found that the pH value decreased and precipitates increased depending on the dose of hafnium chloride. Precipitates were also formed in another study in which hafnium oxide was found to be relatively non-toxic in HaCaT skin cells [15]. We can conclude that hafnium alloys have a tendency to form precipitates.

Individuals with low sperm motility and viability values are considered to have poor semen quality. Poor semen quality negatively impacts reproductive potential, increasing the need for assisted reproductive technologies. To increase the success of assisted reproduction techniques, we also need motile sperm [36]. In addition to the decrease in the number of motile sperm in the HfCl_4_ group, there was also a decrease in viability values. If there was no decrease in viability values, we could have mentioned that hafnium directly affected only sperm movement, that is, the structure of the sperm tail. However, it also showed its toxic effect on viability. When the percentage of viable spermatozoa decreases, the percentage of non-motile spermatozoa increases [37]. However, the increase in the rate of non-motile sperm may not affect the viability of the sperm at the same rate. Clinically, it is also important whether the non-motile sperm is viable or not. The viability rates decreased significantly in all hafnium groups, most notably in the 4 mg/mL HfCl_4_ group at the 40th minute.

Two general approaches can be taken to reduce exposure to the toxicity of heavy metals, especially hafnium. First, detect the presence of exposure. If the presence of exposure is not detected, no action can be taken against the exposure, and thus there is no reduction in exposure. A second approach is to identify the source of the toxic substance. This requires knowledge of the substances used in the manufacture of the products or contained in the products. During the construction or use of the products, there may be a spread to the environment in the form of leakage. In order to detect and prevent this, it is necessary to develop new technologies to improve air and water quality. Thus, exposures can be reduced or even eliminated.

## Conclusions

Although there are studies indicating that hafnium alloys are biocompatible, in our study, we found that HfCl_4_ has negative effects on sperm motility and viability as the dose increases in vitro. These effects may be due to the presence of an acidic environment. More studies are needed to better understand the effect of hafnium on sperm parameters.
